# Point-of-care Ultrasound for Long Head of the Biceps Tendon Rupture

**DOI:** 10.5811/cpcem.2020.7.47777

**Published:** 2020-07-20

**Authors:** Browning S. Wayman, Ryan Joseph

**Affiliations:** University of Texas Health Science Center at San Antonio, Department of Emergency Medicine, San Antonio, Texas

**Keywords:** Biceps tendon rupture, ultrasound, musculoskeletal

## Abstract

**Case Presentation:**

We present a case of a 59-year-old male who presented to the emergency department with left upper arm pain that started suddenly after lifting some plywood a few days prior. Point-of-care ultrasound (POCUS) was performed, which revealed a rupture of the long head of the biceps tendon.

**Discussion:**

Biceps tendon rupture is a relatively rare occurrence; however, rupture of the long head is more common than the short head. Being competent in bedside musculoskeletal POCUS is important for the emergency physician and can help expedite care in cases such as the one presented here.

## CASE PRESENTATION

**Image 1:**
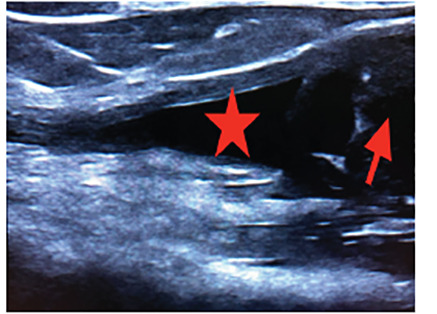
Point-of-care ultrasound in the longitudinal view. The star indicates fluid collection, where the proximal long head biceps tendon (LHBT) is normally seen. The arrow indicates the retracted portion of the LHBT indicating rupture.

**Image 2:**
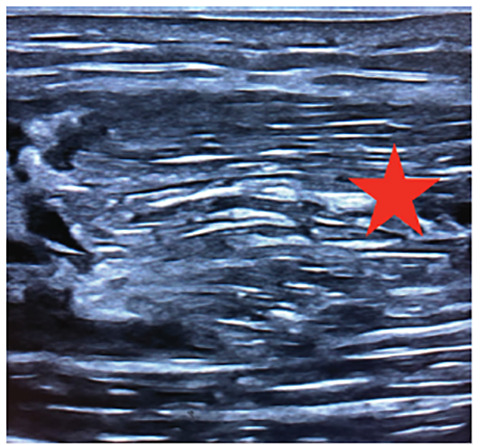
Point-of-care ultrasound in the longitudinal view of the mid long head biceps tendon (LHBT). The star indicates the fluid-filled area where the LHBT should be located with small, echogenic blood clots throughout, indicating a tendon rupture.

**Image 3:**
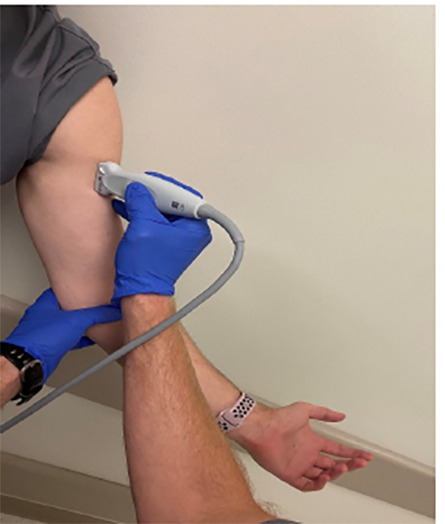
This is the proper transducer orientation for the longitudinal view of the biceps tendon.

A 59-year-old male presented to the emergency department with left upper arm pain, which began abruptly while lifting some plywood about six days prior. On examination, he had significant tenderness at the proximal biceps and significant pain with passive range of motion as well as three out of five strength. There was also a large, soft tissue defect at his proximal bicep with ecchymosis. Point-of-care ultrasound (POCUS) revealed a proximal rupture of the long head of the biceps tendon (LHBT) (Images 1–3).

## DISCUSSION

Biceps tendon rupture is a relatively rare occurrence with a reported incidence rate of 0.53/100,000 over a period of five years, with a male to female ratio of 3:1.^1^ These injuries are more likely to occur in middle age, and associated risk factors include smoking, corticosteroids, overuse, and diabetes. Proximal biceps tendon rupture is more common than distal and usually occurs at the tendon labral junction or the bony attachment.^2^ Also, rupture of the LHBT is far more common than rupture of the short head.^3^

Musculoskeletal ultrasound enables the clinician to perform a dynamic exam at bedside and has a sensitivity and specificity of 88% and 98%, respectively.^4^ In this case, the emergency physician was able to diagnose a complete proximal LHBT rupture via clinical exam and confirmation with POCUS.

**CPC-EM Capsule**

What do we already know about this clinical entity?

Biceps tendon rupture is an uncommon injury that usually occurs when the long head biceps tendon is torn. It has traditionally been a clinical diagnosis.

What is the major impact of the image(s)?

These ultrasound images will aid in identifying the anatomy of a ruptured long head of the biceps tendon.

How might this improve emergency medicine?

Familiarity with musculoskeletal ultrasound can hasten diagnosis in the emergency department and appropriate follow-up.
